# Anti-Atopic Dermatitis Effect of TPS240, a Novel Therapeutic Peptide, via Suppression of NF-κB and STAT3 Activation

**DOI:** 10.3390/ijms242115814

**Published:** 2023-10-31

**Authors:** Dongwoo Lee, Jeon Hwang-Bo, Karpagam Veerappan, Hyunhye Moon, Junhyung Park, Hoyong Chung

**Affiliations:** ANDI Center, 3BIGS Co., Ltd., Hwaseong 18469, Republic of Korea; dwlee@3bigs.com (D.L.); jhwangbo@3bigs.com (J.H.-B.); karpagam@3bigs.com (K.V.); hhmoon@3bigs.com (H.M.); jhpark@3bigs.com (J.P.)

**Keywords:** TPS240, peptide drug, atopic dermatitis, HaCaT cells, anti-inflammatory

## Abstract

Atopic dermatitis (AD) is a relapsing skin disease with persistent inflammation as a causal factor for symptoms and disease progression. Current therapies provide only temporary relief and require long-term usage accompanied by side effects due to persistent relapses. A short peptide, TPS240, has been tested for its potential to subside AD. In this study, we confirmed the anti-atopic effect of TPS240 in vivo and in vitro using a DNCB-induced AD mouse model and TNF-α/IFN-γ-stimulated HaCaT cells. In the AD mouse model, topical treatment with TPS240 diminished AD-like skin lesions and symptoms such as epidermal thickening and mast cell infiltration induced by DNCB, similar to the existing treatment, dexamethasone (Dex). Furthermore, skin atrophy, weight loss, and abnormal organ weight changes observed in the Dex-treated group were not detected in the TPS240-treated group. In TNF-α/IFN-γ-stimulated HaCaT cells, TPS240 reduced the expression of the inflammatory chemokines CCL17 and CCL22 and the pruritic cytokines TSLP and IL-31 by inhibiting NF-κB and STAT3 activation. These results suggest that TPS240 has an anti-atopic effect through immunomodulation of AD-specific cytokines and chemokines and can be used as a candidate drug for the prevention and treatment of AD that can solve the safety problems of existing treatments.

## 1. Introduction

Skin is an effective frontline barrier protecting against pathogens and environmental insults. Given its role as a physical barrier, it is well harbored with defensive inflammatory machinery along with the skin microbiome. The structural disruption of this mechanical barrier due to the interplay of genetic or environmental stimuli results in one of major inflammatory skin diseases, atopic dermatitis (AD). Global prevalence of AD is on the rise, majorly affecting infants (20–30%) and children (15–25%) compared to adult populations (5–10%) [1,2,3]. Although 40–70% of childhood occurrence resolves by adolescence, the disease may persist for a long period, as it has a cycle of relapsing–remitting with recurring acute flare ups. The complex pathogenesis of this disease is a vicious cycle of immune dysregulation and epidermal-barrier dysfunction. Managing AD is highly diverse because of the variability of age in AD patients, diverse AD phenotypes, complexity of the disease itself, and unpredictable flare ups [4,5]. Although recent developments of new drug entities have been added to the current AD therapeutic regimen, the benefit-risk ratio still requires a new drug to manage.

AD is an inflammatory disease caused by an imbalance between T-helper 2 cells (Th2) and T-helper 1 cells (Th1), with Th2 predominant in the acute phase and Th1 involvement in the chronic phase. Th2 activated by antigen challenge secretes many cytokines and chemokines [6]. CCR4 is the major chemokine receptor on Th2 cells. CCL17 and CCL22, the ligands of CCR4, are chemokines that attract Th2 cells to inflamed tissues and are responsible for AD initiation [7]. Thymic stromal lymphopoietin (TSLP) and IL-31 are Th2 cytokines that induce pruritus by activating cutaneous somatosensory neurons either directly or indirectly through the stimulation of immune cells. They are highly expressed in activated mast cells and AD skin, causing allergic inflammation. These are also representative atopic cytokines that promote pathological progression, such as breaking down the skin barrier, worsening itching, and amplifying inflammation [8]. Therefore, CCL17, CCL22, TSLP, and IL-31 are key mediators of inflammatory skin diseases such as AD, and targeting the production of these mediators is considered an important potential treatment for AD.

Therapies aimed at treating AD target multiple components of this disease, primarily defective epidermal barriers, distorted skin microbiome, dysfunctional immune regulation and the resulting itch-scratch cycle [9,10]. Multiple immune pathways are involved in AD, which offer a wider range of pharmacological targets to identify novel anti-inflammatory drugs for treating acute flares as well as long-term disease-modifying treatments. Use of topical corticosteroids, a systemic immune suppressant (cyclosporine), and the recent approval of first-in-class biologics dupilumab (anti-IL-4R), tralokinumab (anti-IL-13), and broad-range small molecule JAK inhibitors (abrocitinib and upadacitinib) for the treatment of moderate to severe AD prove the efficacy of immune-targeted therapies [11,12,13]. However, long-term use of these drugs may cause various side effects, such as skin atrophy, bleeding, vasodilation, and organ toxicity. Therefore, a safe treatment without side effects is required even after long-term use. Peptide drugs have received attention in many therapeutic approaches in recent years due to their efficacy, low synthesis cost, and the fact that they are less immunogenic than high molecular weight biological drugs. Peptide drugs are gaining momentum as antimicrobials and anti-inflammatory modulators for several diseases, notably inflammatory bowel disease, autoimmune diseases, neurological diseases, and tumors [14,15]. Previously, peptides such as AES16-2M, human β-defensins, collagen tripeptide, and parnassin showed anti-inflammatory effects and attenuated AD-like symptoms [16,17,18,19,20].

In our previous study, we reported that parnassin, a novel peptide curated from *Parnassius bremeri* (*P. bremeri*, red-spotted Apollo butterfly), is effective in improving atopic dermatitis [20]. For further study, modifications were made to increase the activity of parnassin. Peptide properties such as cationicity, hydrophobicity, and amphipathicity affect their activity [21,22]. Therefore, we have modified two of the four amino acids of parnassin to increase cationicity and hydrophobicity. Totally, 13 peptides were synthesized and tested for the possibility of suppressing the expression of TSLP, IL-31, CCL17, and CCL22 in HaCaT cells. Among them, TPS240 was selected as a peptide capable of improving atopic dermatitis without cytotoxicity. In this study, we have investigated the anti-AD effect and action mechanism of TPS240 on TNF-α/IFN-γ-stimulated HaCaT cells and in a DNCB-induced AD animal model.

## 2. Results

### 2.1. TPS240 Alleviates AD Symptoms in a DNCB-Induced Mouse Model

**Figure 1:**
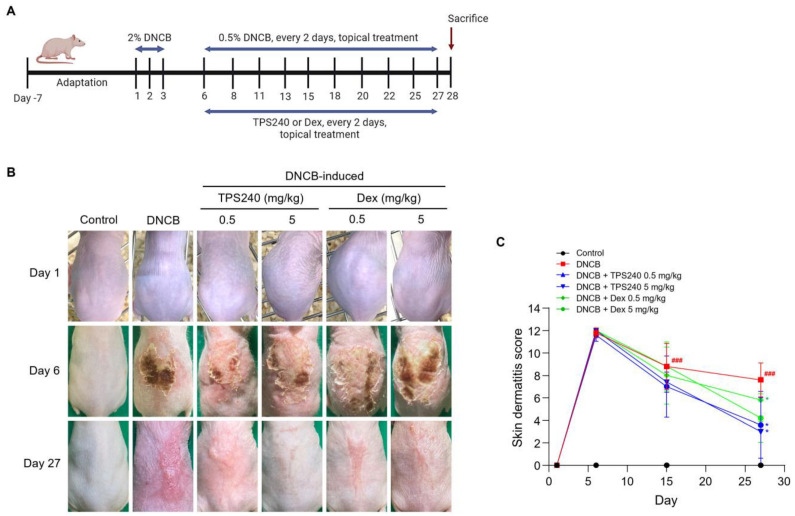
Effects of TPS240 on a DNCB-induced AD mouse model. (**A**) Schematic diagram of the experimental design. AD-like skin lesions were induced by treating mice with 2% DNCB for 3 days. One week after induction, a topical application of 0.5% DNCB was used every 2 days along with TPS240 and Dex to treat the lesion site. All mice were euthanized on day 28. (**B**) Clinical severity of inflammatory skin lesions. Photographs were taken on day 1, 6, and 27. (**C**) Skin dermatitis scores were assessed on days 1, 6, 15, and 27. Data are presented as a mean ± SD (^###^ *p* < 0.001 vs. control group; * *p* < 0.05 vs. DNCB group).

To evaluate the efficacy of TPS240 in alleviating the skin lesions observed in AD, we used a DNCB-induced AD-like contact dermatitis model. The dorsal skin of the hairless BALB/c mice was topically exposed to 2% DNCB for 3 days, and the appearance of AD-like skin damage was confirmed on day 6. TPS240 was topically applied at concentrations of 0.5 mg/kg and 5 mg/kg along with a reduced dose of DNCB (0.5%) every other day for 27 days. Dex was used as a positive control at 0.5 and 5 mg/kg concentrations. The disappearance of redness, inflammation, and desquamation was clearly visible in the peptide treatment group compared with the DNCB group (Figure 1B,C). A notable recovery from DNCB-induced skin damage was observed in the TPS240-treated group at a concentration of 5 mg/kg (Figure 1B,C).

**Figure 2:**
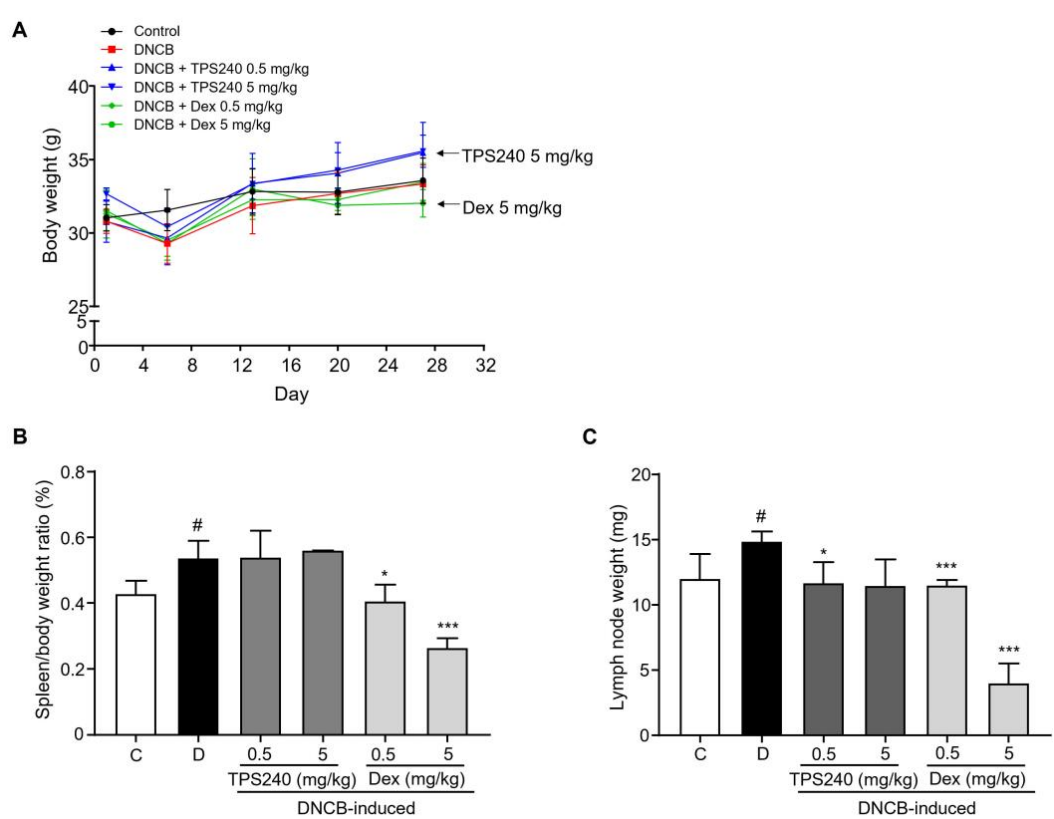
Effect of TPS240 on body weight and organ weight of DNCB-induced AD mouse model. (**A**) Body weights of all mice were measured on day 1, 6, 15, 22, and 27. (**B**,**C**) Spleens and lymph nodes from each group of mice were collected and weighed. The spleen/body weight ratio and lymph node weight of each group are represented as a bar diagram. Data are presented as a mean ± SD (^#^ *p* < 0.05 vs. control group; * *p* < 0.05, *** *p* < 0.001 vs. DNCB group).

Differences in body, spleen, and lymph node weight were examined in all the tested groups. No significant change in body weight was observed in the control, DNCB, and TPS240-treated groups. However, the 5 mg/kg Dex-treated group showed a body weight loss (Figure 2A). DNCB-induced immune response was perceived in immune organs, spleen and lymph nodes. We noticed an increased spleen and lymph node weight in the DNCB-treated group compared with the control group (Figure 2B,C). The TPS240-treated group showed a reduced lymph node weight induced by DNCB, whereas no significant change was observed in the spleen/body weight ratio between DNCB- and TPS240-treated groups. Dex treatment showed a decrease in spleen and lymph node weights induced by DNCB at all tested concentrations. However, at a concentration of 5 mg/kg Dex, abnormal shrinkage of the organs was observed compared with the control group (Figure 2B,C).

### 2.2. TPS240 Inhibits Epidermal Hyperplasia and Mast Cell Infiltration in DNCB-Induced AD-Like Skin Lesions

**Figure 3:**
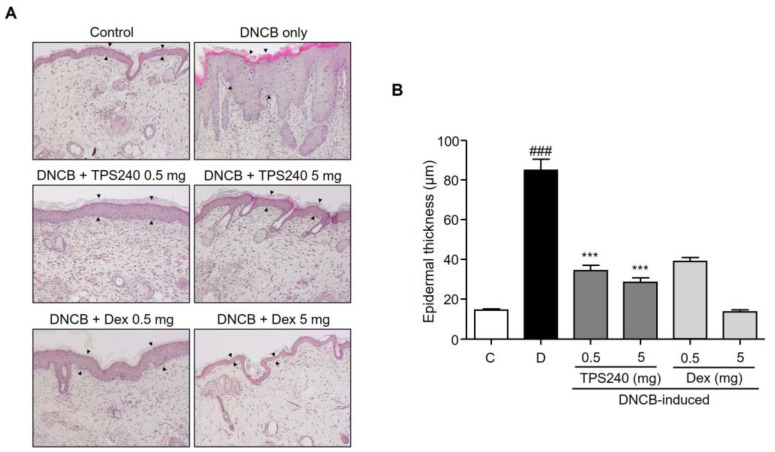
Effect of TPS240 on epidermal thickness in DNCB-induced AD-like skin. (**A**) Mouse skin lesions were fixed with 10% neutral buffered formalin, sectioned to a thickness of 5 μm, and stained with hematoxylin and eosin. All stained sections were digitalized, and images were captured under 400× objective magnification. Scale bar = 100 μm. (**B**) Epidermal thickness was measured using ImageJ software. Data are represented as a bar diagram. (^###^ *p* < 0.001 vs. control group; *** *p* < 0.001 vs. DNCB group).

Skin thickening-associated histological changes of epidermal hyperplasia and inflammatory cell infiltration are hallmarks of AD skin lesions. To observe the histological effect of TPS240 on skin lesions, skin lesions from each treatment group were stained with hematoxylin and eosin to evaluate histopathological changes. Compared with the control group, the skin section of the DNCB-induced group showed thickening of the epidermis exhibiting epidermal hyperplasia and hyperkeratosis (Figure 3). Topical administration of TPS240 significantly reduced epidermal thickness at both the tested concentrations compared to the DNCB-induced group (Figure 3).

**Figure 4:**
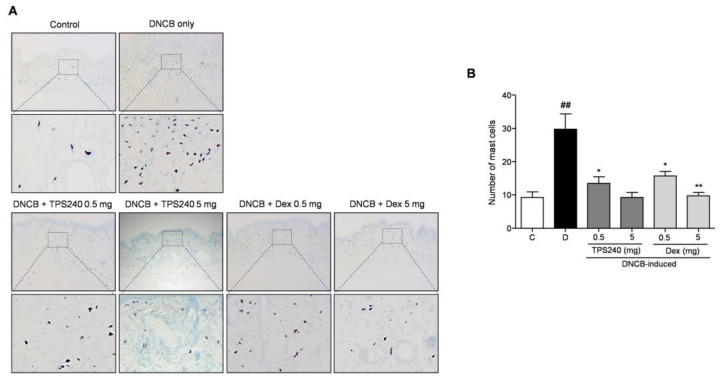
Effect of TPS240 on the mast cell infiltration in DNCB-induced AD-like skin. (**A**) Mouse back skin lesions were fixed with 10% neutral buffered formalin, sectioned to a thickness of 5 μm, and stained with toluidine blue O solution. All stained sections were digitalized, and images were captured. (**B**) Number of mast cells were counted. Data are represented as a bar diagram. (^##^ *p* < 0.01 vs. control group; * *p* < 0.05, ** *p* < 0.01 vs. DNCB group).

Mast cell infiltration, an important inflammatory dysregulation hallmark of AD-linked skin lesions, was examined using toluidine blue O staining. The number of mast cells in the DNCB-induced mice was significantly increased in the lesional skin compared with that in the control group, which was reduced by the topical administration of peptide TPS240 (Figure 4). Decreased epidermal thickness and mast cell infiltration were also observed in Dex-treated mice (Figure 3 and Figure 4). However, the 5 mg/kg Dex-treated group showed an abnormal decrease in epidermal thickness. This indicates the appearance of skin atrophy, which is a typical side effect of the long-term use of topical steroid treatments.

### 2.3. TPS240 Inhibits the Expression of Th2 Responsive Chemokines in TNF-α/IFN-γ-Stimulated HaCaT Cells

**Figure 5:**
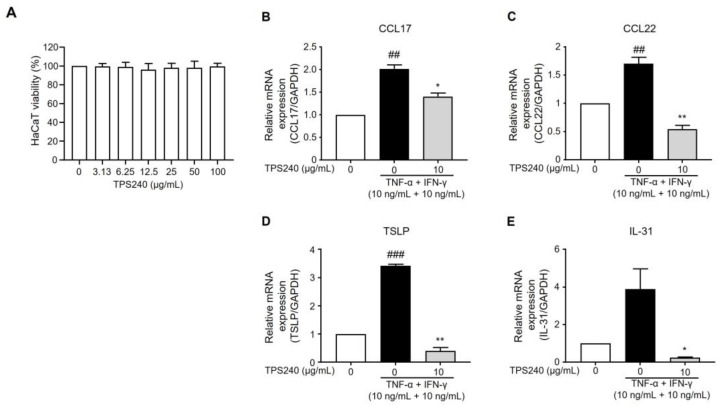
Effect of TPS240 on the mRNA expression of CCL17/TARC, CCL22/MDC, TSLP, IL-31 in TNF-α/IFN-γ-stimulated HaCaT cells. (**A**) HaCaT cells were treated with peptide at the indicated concentrations for 24 h. Cell viability was measured using the WST assay. (**B**–**E**) HaCaT cells were pretreated with 10 μg/mL of peptide for 1 h and treated with TNF-α/IFN-γ for 24 h. Total RNA was isolated, and CCL17, CCL22, TSLP, and IL-31 mRNA levels were analyzed by RT-qPCR using specific primers. GAPDH was used as the internal control. Data are presented as a mean ± SD of three independent experiments. (^##^ *p* < 0.01, ^###^ *p* < 0.001 vs. control; * *p* < 0.05, ** *p* < 0.01 vs. TNF-α/IFN-γ-stimulated HaCaT cells).

Activation of the Th2 pathway is an important hallmark of AD immune signatures. Pro-inflammatory stimuli result in the activation of Th2-responsive chemokines such as CCL17/TARC (thymus and activation-regulated chemokine) and CCL22/MDC (macrophage-derived chemokine) in damaged keratinocytes of AD skin [23,24]. TNF-α/IFN-γ stimulation in HaCaT cells was used to investigate the efficacy of TPS240 in lowering Th2-related chemokine CCL17 and CCL22 expression. The release of cytokines in response to proinflammatory induction was confirmed by the increased mRNA expression of CCL17 and CCL22 in the TNF-α/IFN-γ-treated group. We first confirmed the cytotoxicity of TPS240 using a WST assay in HaCaT cells. As a result of treating HaCaT cells with various concentrations (0–100 μg/mL) of TPS240, it was experimentally confirmed that there was no cytotoxicity of TPS240 at all concentrations up to 100 μg/mL (Figure 5A). TPS240 was pretreated at 10 µg/mL concentration before the induction of TNF-α/IFN-γ (10 ng/mL), and the mRNA expression was quantified using RT-qPCR. In HaCaT cells, TPS240 treatment substantially decreased the expression of CCL17 and CCL22 induced by TNF-α/IFN-γ stimulation (Figure 5B,C). Thus, these results suggest that TPS240 reduced the expression of CCL17 and CCL22, which are Th2-responsive AD-specific chemokines induced by TNF-α/IFN-γ in human keratinocyte cell lines.

### 2.4. TPS240 Reduces the Expression of Pruritus-Related Cytokines in TNF-α/IFN-γ-Stimulated HaCaT Cells

Pruritis is the major deriving factor for the scratch-itch cycle, which exaggerates the skin barrier damage and concurrent inflammatory cascade of events. TSLP, an epidermal alarmin produced in response to proinflammatory stimuli, amplifies pruritus in AD-specific conditions [25,26]. Therefore, we investigated the effect of TPS240 on the expression of TSLP. TSLP mRNA expression was increased in HaCaT cells by TNF-α/IFN-γ stimulation, and the increased expression was reduced by TPS240 treatment (Figure 5D). IL-31 is an “itch cytokine” responsible for epidermal changes and increased infiltration of inflammatory and mast cells in AD skin [27]. Thus, we investigated the effect of TPS240 on the expression of IL-31 in TNF-α/IFN-γ-stimulated HaCaT cells. TNF-α/IFN-γ treatment increased the mRNA expression of IL-31, whereas TPS240 treatment decreased the TNF-α/IFN-γ induced expression of IL-31 in HaCaT cells (Figure 5E). These results suggest that TPS240 exerts antipruritic effects and plays a positive role in AD treatment.

### 2.5. TPS240 Inhibits the Activation of NF-κB in TNF-α/IFN-γ-Stimulated HaCaT Cells

**Figure 6:**
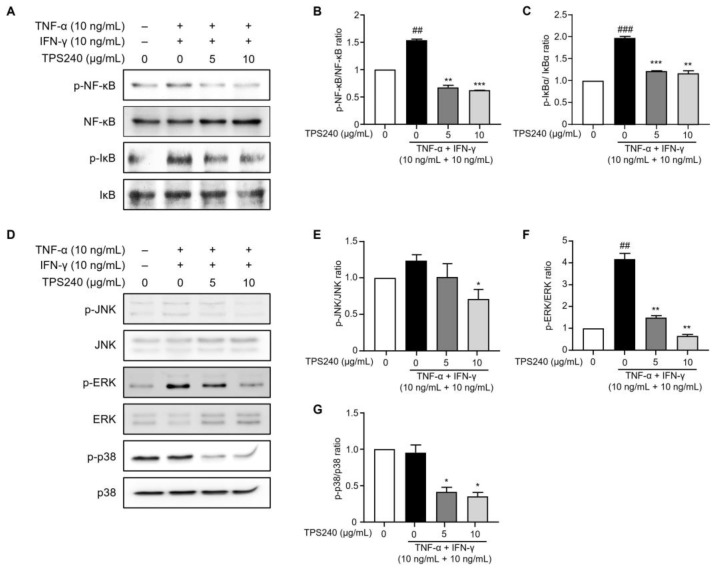
Effect of TPS240 on activation of NF-κB/IκBα and MAPKs in TNF-α/IFN-γ-stimulated HaCaT cells. (**A**,**D**) Cells were pretreated with peptide for 1 h and stimulated TNF-α/IFN-γ for 30 min. Total proteins were subjected to Western blotting using specific antibodies for NF-κB, JNK, ERK, p38, and their respective phosphorylated forms. (**B**,**C**) Relative intensity graphs of total and phosphorylated NF-κB and IκBα. (**E**–**G**) Relative intensity graphs of total and phosphorylated JNK, ERK, and p38. (^##^ *p* < 0.05, ^###^ *p* < 0.01 vs. control; * *p* < 0.05, ** *p* < 0.01, *** *p* < 0.001 vs. TNF-α/IFN-γ-stimulated HaCaT cells).

NF-κB is known to regulate the expression of genes related to allergy, inflammation, and immunity by producing cytokines and chemokines. The nuclear factor NF-κB signaling pathway is considered a prototypical proinflammatory pathway, mainly due to the role of NF-κB in the expression of proinflammatory genes such as adhesion molecules, chemokines, and cytokines [6]. Since nuclear translocation of NF-κB is preceded by IκB phosphorylation and degradation of IκBα, we investigated the effects of TPS240 on phosphorylation of IκBα and NF-κB p65 in TNF-α/IFN-γ-stimulated HaCaT cells. Western blotting results showed that the phosphorylation of IκBα and NF-κB p65 was increased by TNF-α/IFN-γ treatment, whereas TPS240 pretreatment decreased the phosphorylation of IκBα and NF-κB p65 induced by TNF-α/IFN-γ (Figure 6A–C).

### 2.6. TPS240 Inhibits the Activation of JNK, ERK, p38 in TNF-α/IFN-γ-Stimulated HaCaT Cells

MAPKs are specific protein kinases that regulate various cellular processes in response to various external stresses, and major MAPK factors such as JNK, ERK, and p38 are activated in HaCaT cells by TNF-α/IFN-γ stimulation. The expression of inflammatory cytokines is induced by activated MAPKs. To investigate the effect of TPS240 on MAPK activation in HaCaT cells, the phosphorylation of JNK, ERK, and p38—MAPK factors known to be important in atopic dermatitis—was confirmed using Western blotting. In TNF-α/IFN-γ-stimulated HaCaT cells, TPS240 dramatically inhibited the phosphorylation of ERK and p38 compared to the TNF-α/IFN-γ single treatment group, while inhibition of JNK phosphorylation was seen at 10 μg/mL (Figure 6D–G). These results indicate that TPS240 inhibits the activation of JNK, ERK, and p38 following TNF-α/IFN-γ stimulation.

### 2.7. TPS240 Inhibits the Activation of the JAK1/STAT3 Pathway in TNF-α/IFN-γ-Stimulated HaCaT Cells

**Figure 7:**
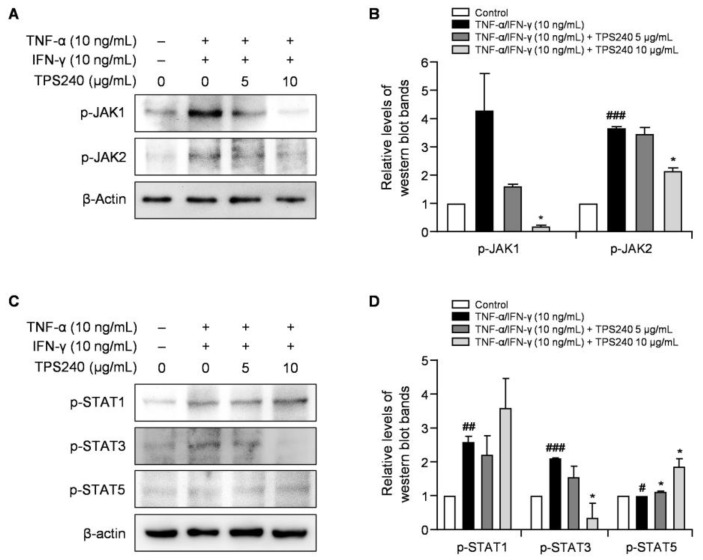
Effect of TPS240 on activation of JAK/STAT pathway in TNF-α/IFN-γ-stimulated HaCaT cells. (**A**,**C**) Cells were pretreated with peptide for 1 h and stimulated TNF-α/IFN-γ for 30 min. Total proteins were subjected to Western blotting using specific antibodies for p-JAK1, p-JAK2, p-STAT1, p-STAT3, p-STAT5, and β-actin. (**B**) Relative intensity graphs of phosphorylated JAK1 and JAK2. (**D**) Relative intensity graphs of phosphorylated STAT1, STAT3, and STAT5. (^#^ *p* < 0.05, ^##^ *p* < 0.01, ^###^ *p* < 0.001 vs. control; * *p* < 0.05 vs. TNF-α/IFN-γ-stimulated HaCaT cells).

The JAK/STAT pathway plays a central role in regulating multiple immune responses involved in the immunopathogenesis of AD. Th2 cytokines, including IL-4, IL-5, IL-13, IL-31, and TSLP, which contribute to the chronic inflammation and pruritus symptoms of AD, are mediated by the JAK-STAT pathway [28]. To investigate the effect of TPS240 on TNF-α/IFN-γ-mediated JAK/STAT pathway activation in HaCaT cells, we confirmed the phosphorylation levels of JAK1, JAK2, STAT1, STAT3, and STAT5 using Western blotting. The phosphorylation of JAK1, JAK2, STAT1, and STAT3 increased in TNF-α/IFN-γ-stimulated HaCaT cells, and the phosphorylation of JAK1 and STAT3 was confirmed to be significantly decreased by TPS240 treatment in a dose-dependent manner (Figure 7). These results indicate that TPS240 inhibits JAK1/STAT3 activation in TNF-α/IFN-γ-stimulated HaCaT cells.

## 3. Discussion

Inflamed and thickened skin is a characteristic lesion in AD, which is a result of pathological inflammatory stimuli and epidermal barrier dysfunction [29]. Thus, to treat AD, we focused on restoring the immune response and preventing skin lesions in the AD mouse model. Topical administration of TPS240 showed anti-inflammatory and anti-AD effects in DNCB-treated mice. AD is a recurring persistent inflammatory skin disease with diverse AD phenotypes and endotypes where the disease itself is significantly different and complex. Although AD is classified as mild, moderate, and severe based on the severity of the disease, the basic need is to treat flared skin in all cases of AD. Emollients are the first therapy to rebuild the skin barrier damage, but this is not sufficient to prolong remission. Topical corticosteroids have been used as anti-inflammatory agents and mainstay drugs for AD. However, long-term and pediatric usage is limited concerning the side effects. In this study, we propose TPS240, a short peptide drug, to treat AD through topical application. Advantages of peptide drugs include target specificity, low immunogenicity, and fast clearance, which qualifies them to be used in long-term disease modifying treatment.

In vivo efficacy of TPS240 was tested using a DNCB-induced AD mouse model. DNCB is used to sensitize the epidermis similar to AD skin lesions to show severe lichen, erythema, blisters, exfoliation, and scaling of the skin. After the topical administration of TPS240, a recovery of skin lesions was clearly visible and consistent till the end of the experiment compared to DNCB-induced mice. The clinical symptoms of AD have been greatly diminished by peptide treatment. The recovery was similar to the standard drug Dex, concluding the potential of TPS240 as an AD drug candidate. Increased lymph node size and weight were due to DNCB treatment; intervention with TPS240 reduced the size and weight, matching the control mice. Abnormal reduction of spleen and lymph nodes was observed in Dex-treated mice, illustrating the side effects of Dex concordant with immune suppressive activity. AD is an inflammatory skin disease characterized by the presence of epidermal layer distribution and immune cell infiltration [30]. Epidermal thickening and mast cell infiltration are pathological hallmarks of AD skin lesions. DNCB induction exhibiting both the hallmarks was visualized by histological analysis and toluidine staining. TPS240 has inhibited both epidermal thickening and mast cell infiltration.

We found that TPS240 shows an anti-inflammatory effect in vitro on the AD cellular model. Human keratinocyte cell line HaCaT stimulated with proinflammatory cytokines TNF-α/IFN-γ was utilized as an in vitro AD model to evaluate the efficacy of TPS240. Stimulation with TNF-α/IFN-γ induces the production of pruritic cytokines and chemotactic chemokines in keratinocytes such as TSLP, IL-31, CCL17/TARC, and CCL22/MDC, further expanding skin inflammation with the recruitment of major T cell lineage, Th2 cells, resulting in epidermal damage [31,32,33]. Thus, we investigated the efficacy of TPS240 in inhibiting AD drivers in TNF-α/IFN-γ stimulated HaCaT cells. In vitro results showed that TPS240 has an anti-inflammatory effect via inhibition of CCL17/TARC, CCL22/MDC, IL-31, and TSLP. The cytokine genes contain different combinations of transcription factor binding elements for NF-κB and STAT. Proinflammatory cytokines such as TNF-α and IFN-γ activate NF-κB and STAT in keratinocytes [7]. Tumor necrosis factor (TNF) is an inflammatory cytokine with many biological functions that correlates various types of tumor cells, including cytostatic and cytotoxic effects, differentiation, and proliferation. TNF-α has been reported to be involved in the generation of CCL17/TARC and CCL22/MDC through the nuclear transcription factor (NF-κB) and mitogen-activated protein kinase (MAPK) signaling pathways. IFN-γ stimulates HaCaT cells and induces the expression of various chemokines and cytokines, such as TARC and MDC, through the JAK/STAT signaling pathway. Additionally, phosphorylation of the Janus kinase/signal transducer and activator of transcription (JAK/STAT) pathway by IFN-γ is known to be an important target for anti-inflammatory mechanisms [6].

To understand the underlying action mechanism of TPS240 in improving skin lesions and inhibiting AD-associated chemokines (CCL17, CCL22) and pruritic cytokines (TSLP, IL-31), we investigated the phosphorylation of MAPK, NF-κB, and JAK/STAT pathway-related proteins using Western blot analysis in TNF-α/IFN-γ-stimulated HaCaT cells. NF-κB plays an important role in the production of pro-inflammatory cytokines such as IL-6, IL-8, IL-1β and chemokines including MDC and TARC [34]. TNF-α is known to degrade IκBα, phosphorylate NF-κB, and translocate it to the nucleus. In this study, we found that TPS240 reduced the expression of NF-κB downstream TARC/CCL17 and MDC/CCL22 genes, so we could hypothesize that TPS240 may interfere with the activation of NF-κB signaling. To verify this, we checked the activation of NF-κB, and we confirmed that TPS240 inhibits phosphorylation of NF-κB. We further identified specific inflammatory signaling pathways that TPS240 can interfere with. MAPKs are serine/threonine-specific protein kinases that respond to a variety of external stresses. The MAPK signaling pathway coordinates multiple cellular processes, including gene expression, cell proliferation, apoptosis, and survival [35]. TNF-α/IFN-γ treatment activates key MAPK factors such as p38, JNK, and ERK in HaCaT cells [34,36]. Inhibition of MAPK is known to reduce the synthesis of intracellular signaling pathways and inflammatory cytokines. Therefore, we further confirmed the possibility of inhibition of MAPK by TPS240. TPS240 inhibited the phosphorylation of MAPKs such as p38, ERK, and JNK. These results show that TPS240 has anti-inflammatory and anti-pruritic effects through inhibition of the NF-κB and MAPK pathways.

We also confirmed that the JAK/STAT signaling pathway is induced by IFN-γ, which is an important factor of inflammation and immune responses. STAT proteins are key components of the signaling pathway for various factors and cytokines [37]. There are seven members of the STAT family, of which STAT1 and STAT3 are responsible for the distribution of IFN-γ through JAK1. Additionally, STAT1 and STAT3 are known to mediate the production of chemokines such as TARC and MDC by TNF-α/IFN-γ stimulation in HaCaT cells [38]. Therefore, we investigated the effect of TPS240 on JAK/STAT pathway activation and confirmed that the increased phosphorylation of JAK1 and STAT3 by TNF-α/IFN-γ was suppressed by TPS240 in HaCaT cells. These results indicate that TPS240 may cure AD targeting JAK1/STAT3.

## 4. Materials and Methods

### 4.1. Peptide Synthesis

TPS240 (KQYR) was synthesized and purified at Lugen Sci Co., Ltd. (Bucheon, Republic of Korea) according to previously described methods [20]. The peptide was dissolved in distilled phosphate-buffered saline (PBS) at a concentration of 1 mg/mL.

### 4.2. Animals

Male SKH-1 mice (BALB/c hairless mice, six weeks old) were purchased from Orient Bio Inc. (Seongnam, Republic of Korea). The mice were maintained at the standard animal facility in an environmentally controlled room with a 12 h light/dark cycle and allowed free access to water. This study (IACUC2204-005, 13 October 2022) was reviewed and approved by the Institutional Animal Care and Use Committee of Woojung Bio (Hwaseong, Republic of Korea). Animal care and experimental procedures followed the guidelines of Woojung Bio for the care and use of laboratory animals.

### 4.3. Establishment of a DNCB-Induced AD-Like Mouse Model

Mice were divided into 6 groups with 5 mice per group: (1) vehicle (control), (2) DNCB, (3) DNCB + TPS240 0.5 mg/kg, (4) DNCB + TPS240 5 mg/kg, (5) DNCB + Dex 0.5 mg/kg, and (6) DNCB + Dex 5 mg/kg. 2,4-Dinitrochlorobenzene (DNCB; Sigma-Aldrich, St. Louis, Mo, USA) was dissolved in the vehicle (3:1, acetone: olive oil) and applied topically on the back skin to induce AD-like skin lesions in mice. As shown in Figure 1A, mice were treated with 100 µL of 2% DNCB at a constant time every day for 3 days. After a week, the appearance of skin lesions was confirmed and treated with DNCB at 0.5% in 100 μL every 2 days along with TPS240 and Dex for 2 weeks. The skin condition was observed weekly and imaged using a digital camera during the experiment. After treatment completion, all mice were euthanized by CO_2_ gas inhalation. Spleens and lymph nodes from each group of mice were collected and weighed. Dorsal skin lesions were collected and fixed with 10% neutral buffered formalin for histological analysis.

### 4.4. Evaluation of Severity of Dermatitis

The dermatitis of each mouse was observed, and the severity of the dermatitis was assessed on days 1, 6, 15, and 27. Dermatitis severity is assessed by four symptoms: (1) erythema/hemorrhage; (2) scarring/dryness; (3) edema; (4) peeling/erosion. The score for each clinical symptom was 0 to 3 (none, 0, mild, 1, moderate, 2, severe, 3), and the total dermatitis score (maximum score 12) was the sum of the individual scores for the four symptoms.

### 4.5. Histological Analysis

The fixed dorsal skin lesions were embedded into paraffin and sectioned at 5 μm thickness. For histopathological observation, paraffin sections were stained with hematoxylin/eosin solution. Infiltrated mast cells were stained using toluidine blue O solution and counted. All sections were digitized under 400× objective magnification, and images were captured. Epidermal thickness was measured using the ImageJ program (NIH, version 1.51j8). Mast cells in toluidine blue-stained sections were counted in three different parts.

### 4.6. Cell Culture

The HaCaT (immortal human keratinocyte) cell line was purchased from Korean Cell Line Bank (Seoul, Republic of Korea) and cultured in Dulbecco’s modified Eagle’s medium (DMEM; WELGENE Inc., Daegu, Republic of Korea) supplemented with 10% heat-inactivated fetal bovine serum (FBS, Corning, NY, USA) and 1% penicillin-streptomycin (Gibco, Grand Island, NY, USA) in a humidified incubator at 37 °C and 5% CO_2_.

### 4.7. Cell Viability Assay

HaCaT cells were seeded at 5 × 10^3^ cells/well in a 96-well plate and incubated for 24 h. Cells were treated with different concentrations (0–100 μg/mL) of TPS240 for another 24 h. Cell viability was measured by WST assay using EZ-Cytox (Dogen, Seoul, Republic of Korea) according to the manufacturer’s instructions. A total of 10 μL of EZ-Cytox was added to each well and the plate was incubated at 37 °C for 3 h. The optical density (OD) was measured at a wavelength of 450 nm using an INNO microplate spectrophotometer (LTek, Seongnam, Republic of Korea). Cell viability was represented as the percentage of live cells in the TPS240-treated group versus the control group.

### 4.8. Real-Time Quantitative PCR (RT-qPCR) Analysis

HaCaT cells (3 × 10^5^ cells/well) were cultured in 6-well plates and treated with 10 ng/mL of recombinant human TNF-α (R&D systems, Minneapolis, MN, USA) and 10 ng/mL of recombinant human IFN-γ (R&D systems, Minneapolis, MN, USA) in the presence or absence of TPS240 (5, 10 μg/mL) for 24 h. Total RNA isolation was performed using RiboEx^TM^ reagent (GeneAll Biotechnology, Seoul, Republic of Korea) according to the manufacturer’s instructions. One μg of total RNA was used for cDNA synthesis with AccuPower^®^ RT-PCR PreMix (Bioneer corporation, Daejeon, Republic of Korea). cDNA was used for the amplification of CCL17, CCL22, TSLP, and IL-31 mRNAs with gene-specific primers (Table 1) using EzAmp FAST qPCR 2X Master Mix (ELPIS Biotech, Daejeon, Republic of Korea) following manufacturer’s protocol. GAPDH mRNA was used as an internal control. The CCL17, CCL22, TSLP, and IL-31 mRNA levels were normalized by GAPDH levels; then, relative mRNA expression was calculated by 2^−ΔΔCt^ method.

### 4.9. Western Blot Analysis

HaCaT cells were pretreated with TPS240 for 1 h and exposed to TNF-α/IFN-γ (10 ng/mL) for 30 min. Cells were harvested and lysed with 200 µL of RIPA buffer (Biosesang, Seongnam, Republic of Korea). Total protein concentrations were quantified using a Bradford assay reagent (Biosesang, Seongnam, Republic of Korea). Equal amounts of total protein (40 µg) were separated using 10% SDS-PAGE and transferred onto Immobilon^®^-P polyvinylidene fluoride (PVDF) membrane (Merck Millipore, St. Louis, MA, USA). Membranes were incubated in blocking solution (3% skim milk in TBS containing 0.05% Tween 20) for 1 h and incubated overnight at 4 °C with primary antibodies (anti-ERK, anti-p-ERK, anti-p38, anti-p-p38, anti-JNK, anti-p-JNK, anti-NF-κB, anti-pNF-κB, anti-IκBα, anti-p-IκBα, anti-JAK1, anti-JAK2, anti-JAK3, anti-pSTAT1, anti-pSTAT3, anti-pSTAT5, anti-GAPDH) (Cell signaling, San Diego, CA, USA) diluted 1:1000 in blocking solution. Then, the membranes were washed with TBS-T and probed with peroxidase-conjugated anti-mouse-IgG and anti-rabbit-IgG antibodies (Cell signaling) at 1:5000 dilution in a blocking solution. Protein bands were detected using SuperSignal West Pico PLUS reagents (Thermo Fisher Scientific, Waltham, MA, USA).

### 4.10. Statistical Analysis

All data were represented as mean ± SD. Student’s *t*-test and one-way ANOVA were used to evaluate the significance between groups (^#^ *p* < 0.05, ^##^ *p* < 0.01, ^###^ *p* < 0.001 vs. control group; * *p* < 0.05, ** *p* < 0.01, *** *p* < 0.001 vs. DNCB group).

## 5. Conclusions

**Figure 8:**
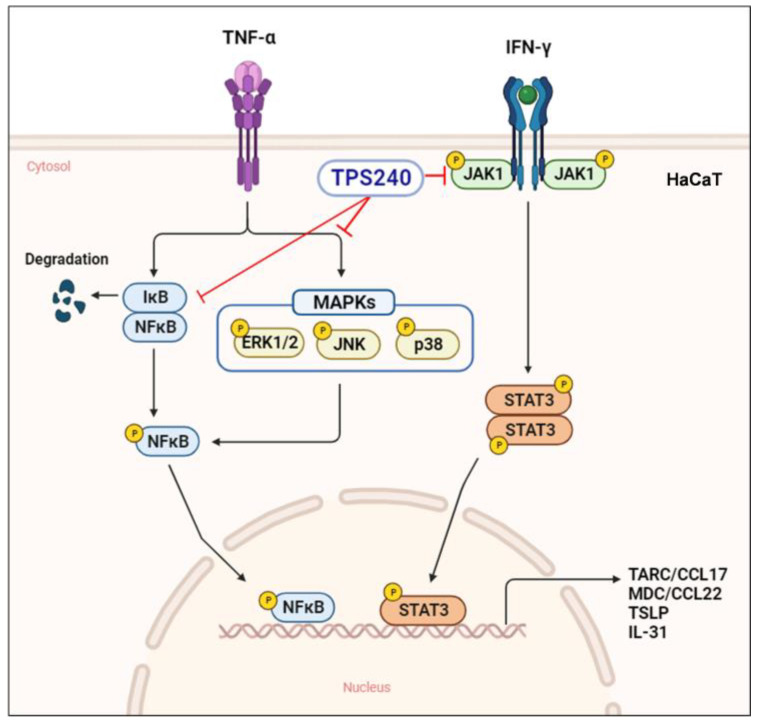
Schematic illustration of the mechanism of action of TPS240.

In conclusion, TPS240 showed similar or comparable therapeutic effects to dexamethasone, one of the existing treatments, without side effects in the DNCB-induced AD-like animal model. In addition, it was confirmed that TPS240 suppresses cytokines and chemokines, which are the final products of inflammation in TNF-α/IFN-γ-stimulated HaCaT cells. We found that the inhibitory effect of TPS240 was due to inhibition of NF-κB and STAT3 by suppressing the MAPKs and JAK1 signaling pathways (Figure 8). Our findings suggest that TPS240 may be a safer and more effective treatment for atopic dermatitis.

## Figures and Tables

**Table 1:** List of RT-qPCR primer pairs.

Genes	Primer Sequences	
CCL17	Forward	5′-ACTGCTCCAGGGATGCCATCGTTTTT-3′
	Reverse	5′- ACAAGGGGATGGATCTCCCTCACTG-3′
CCL22	Forward	5′-AGGACAGAGCATGGCTCGCCTACAGA-3′
	Reverse	5′-TAATGGCAGGGAGGTAGGGCTCCTGA-3′
TSLP	Forward	5′-GGGGCTAAACCATGACAGAA-3′
	Reverse	5′-GTTTGGCTGAAGGCTTGTTC-3′
IL-31	Forward	5′-CGACGTCTGTGCTCTTTCTG-3′
	Reverse	5′-AGCATCTTCGAGAGGGACTG-3′
GAPDH	Forward	5′-GACCCTCGAAATCCCATCACAG-3′
	Reverse	5′-GTGCGAACTTCCACGGTGTGTT-3′

## Data Availability

All data generated or analyzed during this study are included in this published article.
